# Physiological Hypoxia Enhances Stemness Preservation, Proliferation, and Bidifferentiation of Induced Hepatic Stem Cells

**DOI:** 10.1155/2018/7618704

**Published:** 2018-02-13

**Authors:** Xiaosong Zhi, Jun Xiong, Mengchao Wang, Hongxia Zhang, Gang Huang, Jian Zhao, Xiaoyuan Zi, Yi-Ping Hu

**Affiliations:** ^1^Center for Stem Cells and Medicine, Department of Cell Biology, Second Military Medical University, 800 Xiangyin Road, Shanghai 200433, China; ^2^Department of Histology and Embryology, Second Military Medical University, 800 Xiangyin Road, Shanghai 200433, China; ^3^The Third Department of Hepatic Surgery, Eastern Hepatobiliary Surgery Hospital, Second Military Medical University, Shanghai 200433, China; ^4^Shanghai Key Laboratory for Molecular Imaging, Shanghai University of Medicine & Health Sciences, 279th Zhouzhu Road, Shanghai 201318, China

## Abstract

Induced hepatic stem cells (iHepSCs) have great potential as donors for liver cell therapy due to their self-renewal and bipotential differentiation properties. However, the efficiency of bidifferentiation and repopulation efficiency of iHepSCs is relatively low. Recent evidence shows that physiological hypoxia, a vital factor within stem cell “niche” microenvironment, plays key roles in regulating tissue stem cell biological behaviors including proliferation and differentiation. In this study, we found that physiological hypoxia (10% O_2_) enhanced the stemness properties and promoted the proliferation ability of iHepSCs by accelerating G1/S transition via p53-p21 signaling pathway. In addition, short-term hypoxia preconditioning improved the efficiency of hepatic differentiation of iHepSCs, and long-term hypoxia promoted cholangiocytic differentiation but inhibited hepatic differentiation of iHepSCs. These results demonstrated the potential effects of hypoxia on stemness preservation, proliferation, and bidifferentiation of iHepSCs and promising perspective to explore appropriate culture conditions for therapeutic stem cells.

## 1. Introduction

Induced hepatic stem cells (iHepSCs) are lineage-reprogrammed cells originating from murine embryonic fibroblasts via two confirmed transcription factors Hnf1*β*, Foxa3. Our previous work [1] showed that iHepSCs expressed hepatic stem cell markers CK19, EpCAM, Sox9, and Lgr5 and possessed the capability of bipotentially differentiating to mature hepatocytes and cholangiocytes under certain conditions in vitro. In addition, iHepSCs could engraft and save the liver in a mouse model of hereditary tyrosinemia type I (HT1) and also engraft as cholangiocytes into bile ducts of mice with DDC-induced bile ductular injury, showing great perspective for cell therapy of chronic liver injury. However, the induction efficiency of both hepatic and cholangiocytic differentiation and repopulation efficiency of iHepSCs are relatively low.

Physiological hypoxia refers to a condition where the oxygen concentration inside the human body is significantly lower than that in the atmosphere. The oxygen concentration is one of the most important regulators for organ development and tissue construction, with a well-established steering effects, on overall cell metabolism, proliferation, and differentiation [2]. Recent evidence shows that changes in the microenvironment of specific tissue stem cells residing in stem cell “niche,” including the oxygen concentration in particular, play key roles in regulating their biological behaviors [3]. Cells are normally cultured in vitro in the presence of 5% CO_2_ and about 20% oxygen. However, the natural cell microenvironment contains a much lower oxygen concentration ranging from 12% in arterial blood to 1–7% in a variety of other tissues [4]. Studies in recent years have provided evidence regarding the negative influence of the ambient O_2_ concentration on stem cells, causing longer population doubling time and DNA damage [5, 6]. In contrast, 5% O_2_ hypoxia enhanced the growth of dental pulp stem cells (DPSCs) and the stem property of stem cells [7]. Notably, low O_2_ tension promoted the survival of neural crest cells and hematopoietic stem cells and maintained pluripotency of human embryonic stem cells (ESCs) [8, 9]. Hypoxia also promoted reprogramming mouse embryonic fibroblasts into induced pluripotent stem cells and neural progenitors [10, 11].

The liver is the major organ responsible for metabolism. It is constructed by millions of functional units termed hepatic lobules [12]. In each hepatic lobule, the blood inflows from the hepatic capillary artery or portal vein system, making liquid exchanges through sinusoids, and outflows towards the central vein, which results in physiological oxygen gradient from the portal area to the central area. It is reported that the mean oxygen concentration is 9–11% in the periportal area and 5–7% in the perivenous area under physiological conditions [13, 14]. Moreover, adult liver stem cells are recognized to reside in canals of Hering in the portal area [15], but whether the physiological oxygen environment would affect the biological behaviors of liver stem cells remains unknown.

In the present study, we cultured iHepSCs in a 10% O_2_ environment mimicking the physiological hypoxic conditions and found that physiological hypoxia could enhance the stemness properties and promote the proliferation ability of iHepSCs by quickening G1/S transition. In addition, short-term hypoxia preconditioning could improve the efficiency of hepatic differentiation of iHepSCs, and long-term hypoxia could promote Matrigel-induced 3D cholangiocytic differentiation and inhibited hepatic differentiation of iHepSCs. These results are of great significance to understand the effects of hypoxia on biological behaviors of stem cells and have promising perspective to explore appropriate culture conditions for therapeutic stem cells.

## 2. Materials and Method

### 2.1. Cell Culture and Hypoxic Procedures

Mouse induced hepatic stem cells (iHepSCs) were obtained from our own laboratory. The medium for iHepSCs was SCMA as previously described [1] and changed every other day. Cells were cultured under a physiologically hypoxic condition using a gas mixture of 85% N_2_, 5% CO_2_, and 10% O_2_ and maintained in an incubator (Sanyo, MCO-18M, Japan) with two gas sensors (CO_2_ and O_2_). The O_2_ concentration was maintained at the desired level by automatic delivery of pure N_2_ from a N_2_ tank into the incubator to evacuate the excess O_2_. The incubator maintained 37°C temperature similar to the control culture.

### 2.2. Quantitative Real-Time PCR (qRT-PCR)

Total RNA was extracted from cells with TRIzol reagent (Invitrogen, USA), and 2 *μ*g RNA was reversed to cDNA via SuperScript II reverse transcriptase (Invitrogen) according to manufacturer's instructions. All primer sequences were obtained from NCBI database (Supplemental Table 1). qRT-PCR was performed in triplicate for each sample using ABI-7900 (Applied Biosystems, USA) with SYBR Green Premix Ex Taq (Takara). The resulting cDNAs were amplified by a two-step method under the following conditions: 95°C for 5 min as initial denaturation followed by 40 cycles of denaturation at 95°C for 15 sec, annealing combined with extension at 60°C for 30 sec.

### 2.3. Western Blotting Assay

Total protein was extracted from cells with RIPA lysis buffer, supplied with protease inhibitor cocktail, and incubated on ice for 30 min. All cell lysate was cleared by centrifugation (12,000*g* for 15 min at 4°C). The protein concentration of the samples was determined by bicinchoninic acid assay. Proteins were separated on 8% or 12% (determined by protein molecular weight) SDS-polyacrylamide gels and electroblotted onto polyvinylidene fluoride membranes (Millipore). The membranes were blocked with blocking buffer (TBS-Tween containing 5% skim milk) for 1 h at room temperature and then incubated with primary antibodies at 4°C overnight. Then, the membranes were washed for three times with TBS-Tween and incubated with HRP-conjugated secondary antibodies at room temperature for 1 h. Immunoreactive bands were detected by the SuperSignal West Pico Chemiluminescent Substrate (Thermo Fisher).

### 2.4. BrdU Incorporation and Immunofluorescence Staining

The effect of hypoxia on the proliferative activity of iHepSCs was investigated by bromodeoxyuridine (BrdU, Sigma, St. Louis, MO, USA) incorporation. After 24 h incubation under the hypoxic condition, iHepSCs were labeled with 10 M BrdU for 2 h, fixed in 4% paraformaldehyde for 15 min, washed with PBS for 5 min × 3, and incubated in 2 N hydrochloric acid (HCl) for 30 min at 37°C and in 0.1 M sodium borate (pH 8.5) for 10 min (exclusively in BrdU incorporation assay). Cells were washed with PBS-Tween, blocked with 1% bovine serum albumin (BSA) for 30 min at room temperature, and incubated overnight at 4°C with primary antibodies in PBS containing 0.1% Triton X-100 and 1% BSA. After washing in PBS, cells were reacted with the fluorescent-labeled secondary antibody for 1 h at 37°C. The nucleus was counterstained with Hoechst 33342. Images were obtained with a 50i Nikon fluorescence microscope (Nikon). The information about the antibodies is listed in Supplemental Table 2.

### 2.5. Colony-Forming Assay

Single-cell suspension was obtained by EDTA-trypsin digestion and limited dilution. One hundred cells were plated in each 35 mm dish (Corning), fixed with 4% PFA for 15 min at room temperature, stained by crystal violet, and observed under an optical microscope. The number of colonies with more than 50 cells was counted.

### 2.6. Cell Counting Kit 8 Assay

Cell proliferation kinetics was assessed by cell counting kit 8 (CCK8, DOJIMDO). Cells were seeded onto a 96-well plate for 1000 cells per well, and the culture procedure was performed according to manufacturer's instructions.

### 2.7. Cell Cycle Analysis

Flow cytometry was performed to analyze distributions of cell cycle by Becton, Dickinson FACS Aria (BD, Bioscience). Cells were digested to single-cell suspension, fixed with 70% ice-cold ethanol overnight at 4°C, and stained with propidium iodine (50 ng/ml, BD Biosciences) for 10 min at room temperature. Cell cycle distributions were analyzed and fitted by FlowJo 10.

### 2.8. In Vitro Differentiation

Hepatocyte differentiation was induced by switching the medium to basal SCMA supplemented with 20 ng/ml HGF (R&D system), 20 ng/ml Oncostatin M (OSM, R&D system), 0.1 *μ*M dexamethasone (Dex, Sigma-Aldrich), and with 2 *μ*M TGF*β* receptor inhibitor (E-616452) or *γ*-secretase inhibitor (Compound E, 0.5 and 1 *μ*M) for 2 weeks. The hepatic differentiation was verificated by glycogen storage assay using a periodic acid-Schiff staining kit (Sigma-Aldrich) according to the product instructions. Bile duct differentiation was induced by 3D culture. iHepSCs were suspended in cold SCMA supplemented with 20 ng/ml EGF and mixed 1 : 1 with Matrigel. Then, the mixture was plated into 24-well plate (0.5 ml/well) and placed in an incubator at 37°C for 30 min to allow the formation of 3D Matrix. 0.5 ml of SCMA was then carefully overlayed on the gel. The cells were cultured for approximately 1 week to allow the formation of bile duct-like structures [16].

### 2.9. Enzyme-Linked Immunosorbent Assay (ELISA)

After iHepSCs were hepatic-induced for 15 days [16], the medium was collected. The mouse albumin ELISA (Abcam) was performed according to manufacturer's instruction.

### 2.10. Statistical Analysis

All experiments were performed for at least triple biological replicates. Data were reported as mean ± SEM. Statistical analysis and diagrams were carried out with GraphPad Prism 5.0. Significance of results was analyzed using Student's *t*-test or one-way ANOVA, followed by Dunnett's test procedure for multiple comparisons with the appropriate control. *p* < 0.05 was considered statistically significant.

## 3. Results

### 3.1. Physiological Hypoxia Enhances the Stemness Properties of iHepSCs

**Figure 1:**
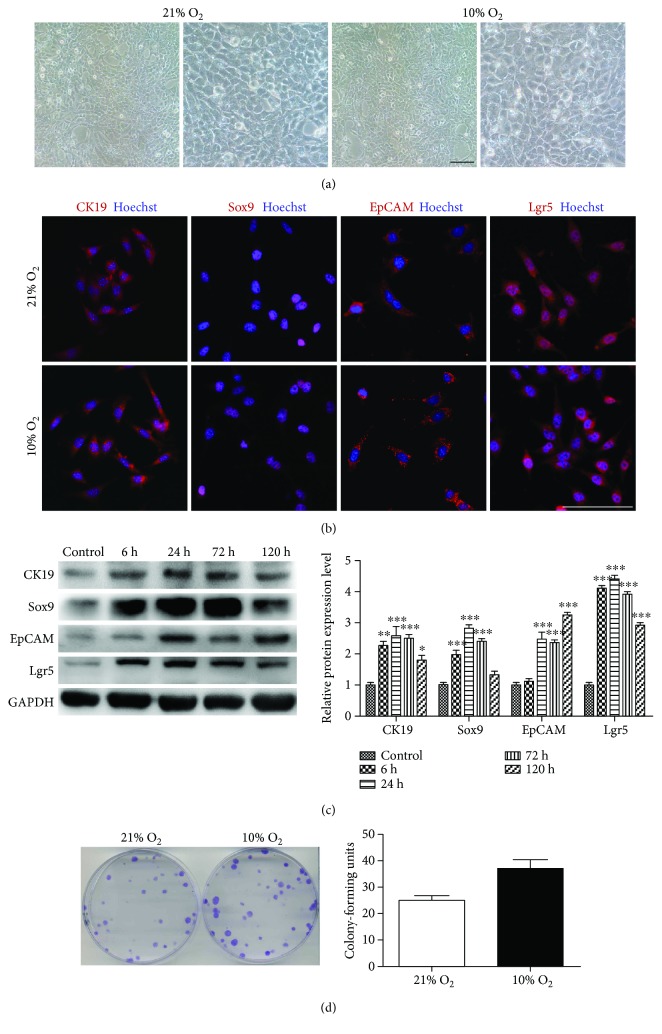
The expression of hepatic stem cell markers of iHepSCs under hypoxia environment. (a) Morphology of iHepSCs under the bright field in each group. (b) Immunofluorescence staining: expression of CK19, Sox9, EpCAM, and Lgr5 of iHepSCs cultured in normoxia and physiological hypoxia environments; nuclei counterstained with Hoechst 33342. (c) Western blot assay and quantitative analysis: expression of CK19, Sox9, EpCAM, and Lgr5 in iHepSCs under normoxia and hypoxia for 6, 24, 72, and 120 h, respectively. (d) Colony-forming units and quantitative analysis in each group. Statistical significance: ^∗^ *p* < 0.05, ^∗∗^ *p* < 0.01, and ^∗∗∗^ *p* < 0.001. Scale bars = 100 *μ*m.

Knowing that low oxygen tension preserved stemness of bone mesenchymal stem cells (BMSCs), adipose-derived MSCs (ADMSCs), and multiple cancer cells [17–19], we predicted that hypoxia may preserve the stem properties of iHepSCs. It was found that iHepSCs cultured in hypoxia morphologically showed a typical epithelial-like phenotype with a high nucleocytoplasmic ratio similar to normoxia-cultured iHepSCs (Figure 1(a)), indicating that iHepSCs maintained the basic stem cell phenotype. Subsequent detection of the expression of the specific liver stem cell markers CK19, Sox9, EpCAM, and Lgr5 of hypoxia-cultured iHepSCs via immunofluorescence staining showed that almost all iHepSCs expressed these markers (Figure 1(b)). To investigate the dynamic changes of the expression level of these stem cell markers during the hypoxia-disposing process, the related proteins were analyzed by Western blot assay. It was found that the expressions of all these stem cell markers in hypoxia-cultured iHepSCs were significantly higher than those in the control group and reached the peak at 24 h after hypoxia culture (Figure 1(c)). To further elucidate their stem properties and single-cell viability, single-cell colony-forming assay was performed. It was found that hypoxia-cultured iHepSCs formed more colony units as compared with the control group (37.0 ± 3.512 versus 25.3 ± 1.453) (Figure 1(d)). These results suggest that iHepSCs enhanced the stemness properties in the physiologic hypoxia environment.

### 3.2. Physiological Hypoxia Promotes the Proliferation Ability of iHepSCs

**Figure 2:**
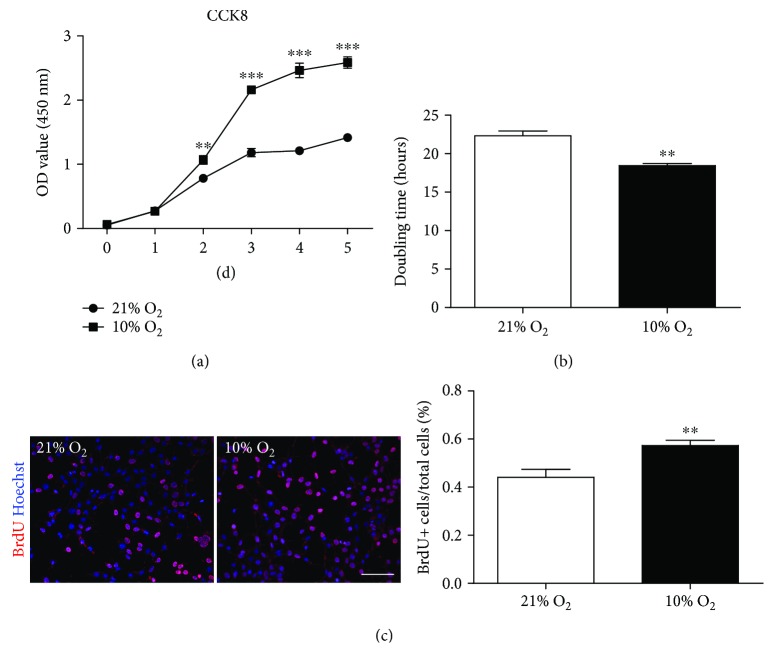
Physiological hypoxia promotes the proliferation ability of iHepSCs. (a) CCK8 assay: proliferation kinetics of iHepSCs cultured under normoxia and hypoxia. (b) Cell doubling time in each group. (c) BrdU incorporation and quantitative analysis: ratio of BrdU-positive cells to total cells in each group. Statistical significance: ^∗∗^ *p* < 0.01 and ^∗∗∗^ *p* < 0.001. Scale bars = 100 *μ*m.

Enhanced stemness properties were commonly accompanied with the increased proliferation ability and rate, while attenuated stemness resulted in diminished cell proliferation, whether in tumor stem cells or tissue stem cells [20–23]. To investigate whether enhancement of the stemness properties would affect the proliferation ability of iHepSCs, BrdU labeling, population doubling time assay, and CCK-8 assay were performed. The CCK-8-assay showed that the proliferation rate was faster in iHepSCs cultured in hypoxia than those cultured in normoxia, which was of statistical significance just from the 2nd day (Figure 2(a)). The population doubling time in iHepSCs cultured in hypoxia is nearly 17% shorter than that in control culture group (22.30 ± 0.643 versus 18.43 ± 0.290) (Figure 2(b)). BrdU incorporation assay indicated that the ratio of proliferated hypoxia-cultured iHepSCs reached 0.57 ± 0.022 versus 0.44 ± 0.033 in the control group (Figure 2(c)). These results implicate that physiological hypoxia could increase the proliferation ability of iHepSCs.

### 3.3. Physiological Hypoxia Accelerates G1/S Transition of iHepSCs via p53-p21 Signaling Pathway

**Figure 3:**
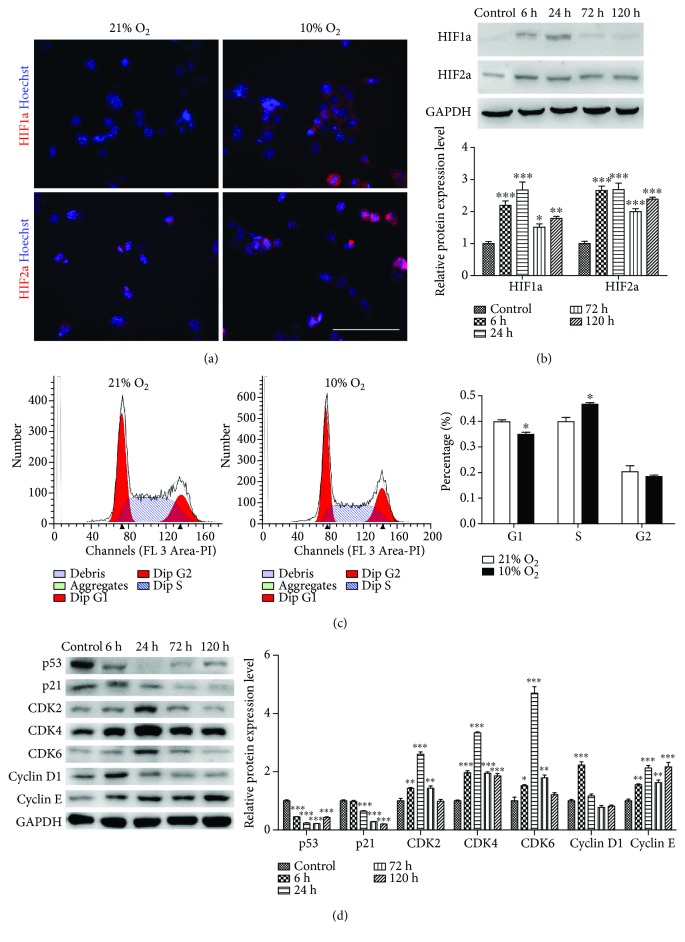
Physiological hypoxia promotes G1/S phase transition in iHepSCs. (a) Immunofluorescence staining: expression of HIF1a and HIF2a in each group; nuclei counterstained with Hoechst 33342. (b) Western blot assay and quantitative analysis: expression of HIF1a and HIF2a in iHepSCs cultured under normoxia and hypoxia for 6, 24, 72, and 120 h, respectively. (c) Flow cytometry and quantitative analysis of cell cycle distributions in each group. (d) Western blot assay and quantitative analysis: expression of cell cycle-related protein of iHepSCs cultured in normoxia and hypoxia for 6, 24, 72, and 120 h, using GAPDH as the internal standard. Statistical significance: ^∗^ *p* < 0.05, ^∗∗^ *p* < 0.01, and ^∗∗∗^ *p* < 0.001. Scale bars = 100 *μ*m.

The hypoxia-inducible factors (HIFs) are a family of heterodimeric transcription factors that act as main regulators of homeostatic transcriptional response to hypoxia in virtually all cells. Active HIFs are composed of alpha and beta subunits. Three alpha subunits (HIF1a, HIF2a, and HIF3a) all bind to a common b subunit HIF1b. HIF1a and HIF2a are two major indexes for hypoxia in humans, mice, and rats [24]. iHepSCs also showed HIF1a and HIF2a expression under hypoxia (Figure 3(a)). Besides, the expression of HIF1a was remarkably and significantly increased under hypoxia within 24 h and then dropped down, while the expression of HIF2a consistently remained at a high level (Figure 3(b)). These results are consistent with a prior report that HIF1a expression could represent an acute response to low pO_2_, whereas HIF2a level may increase over time in hypoxia and play a role during chronic hypoxia [25].

Hypoxia could contribute to the proliferation of cancer cells by promoting G1/S transition [26, 27]. To test whether the enhanced proliferating effect of hypoxia on iHepSCs was attributed to the similar mechanism, we performed cell cycle distribution analysis by flow cytometry. Compared with the control group, hypoxic treatment for 24 h decreased the proportion of cells in the G1 phase (from 0.399 ± 0.017 to 0.467 ± 0.006) and increased the proportion of cells in the S phase (from 0.398 ± 0.008 to 0.349 ± 0.008) (Figure 3(c)). To further validate the underlying molecular changes for G1/S, we detected protein changes in p53-p21 signaling, knowing that it acts as a main regulator of G1/S transition by two downstream effectors, including CDK4/6-CyclinD and CDK2-CyclinE kinase complexes [28, 29]. The results showed that the expression of both p53 and p21 was decreased under hypoxia. Unlike p53 and p21, the positive regulators (CDK2, CDK4, and CDK6) all increased under hypoxia, reaching the highest level after hypoxia for 24 h and then dropped down (Figure 3(d)). In addition, cyclinD1 was upregulated only at 6 h and then returned to the normal level. However, CyclinE remained at a high expression level during the whole hypoxic process (Figure 3(d)). These results probably indicate that CyclinD1 and CyclinE functioned in different ways. The above data suggest that hypoxia triggered G1/S phase transition in iHepSCs via p53-p21 signaling.

### 3.4. The Effects of Physiological Hypoxia on Bipotential Differentiation of iHepSCs

**Figure 4:**
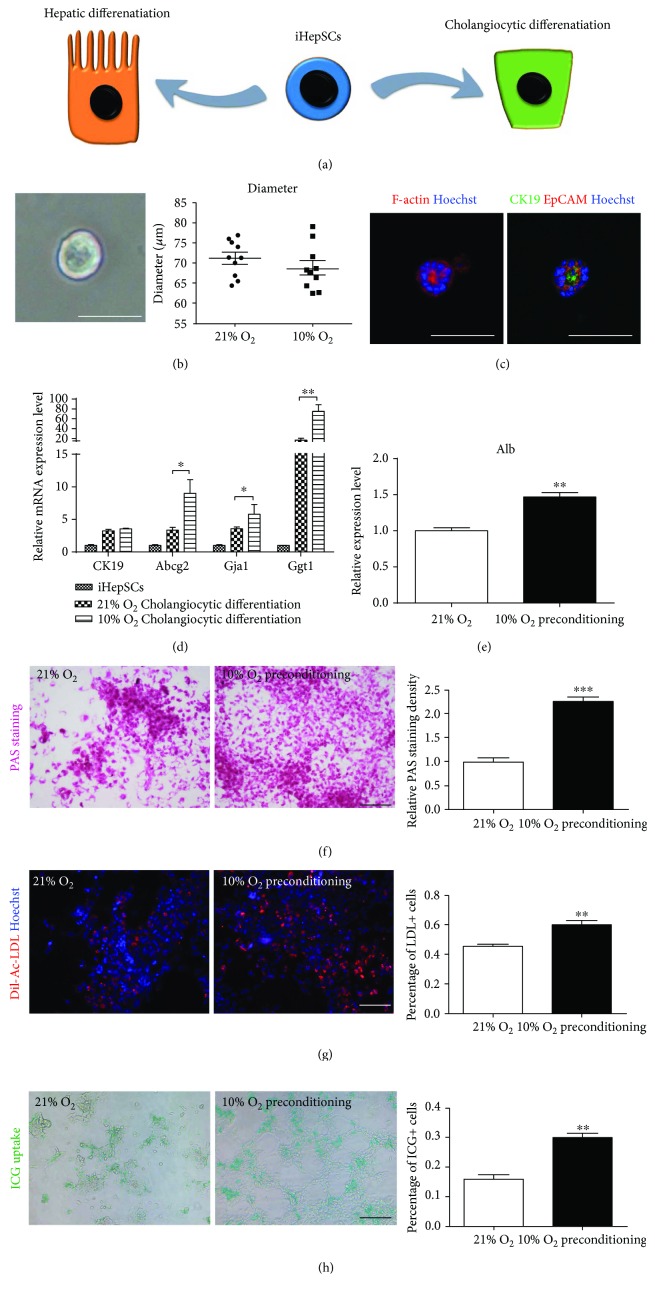
Effects of hypoxia on bipotential differentiation of iHepSCs in vitro. (a) Diagrams showing differentiation of iHepSCs into hepatocytes and cholangiocytes. (b) Morphology of the typical cystic tube structure after induction of iHepSCs to cholangiocytes under hypoxia and their diameter in each group. (c) Immunofluorescence staining: expression of F-actin, CK19, and EpCAM in the cystic tube. (d) qRT-PCR: relative expression of biliary markers in iHepSCs, iHepSCs induced under normoxia, and those induced under hypoxia. (e) ELISA: expression of albumin in the secretion in each group. (f–h) PAS staining, DiI-ac-LDL uptake, and indocyanine green (ICG) uptake and their quantitative analysis: characteristics of mature hepatocytes in each group. Statistical significance: ^∗^ *p* < 0.05, ^∗∗^ *p* < 0.01, and ^∗∗∗^ *p* < 0.001. Scale bars = 100 *μ*m.

iHepSCs could be induced into mature hepatocytes and cholangiocytes under certain conditions (Figure 4(a)) according to the previous study [1]. Hypoxia was reported to promote the efficiency of chondrogenic differentiation in human mesenchymal stem cells and also endowed neural progenitors with enhanced dopaminergic differentiation [30, 31]. So, we hypothesized that hypoxia may be able to promote differentiation of iHepSCs towards hepatocytes and cholangiocytes.

In the hypoxic cholangiocytic induction, iHepSCs also formed cystic duct structures and strongly expressed biliary markers CK19, EpCAM, as well as F-actin (a polarity index) (Figures 4(b) and 4(c)), similar to the previous study [1], but the size was not statistically different from that in the control culture group (Figure 4(b)). qRT-PCR analysis showed that the expression of cholangiocyte-related genes Abcg2, Gja1, and Ggt1 was higher in hypoxic induction than that in normal induction (Figure 4(d)). However, the expression of hepatic hepatocyte-related genes Hnf1a, Alb, and Afp in hypoxic induction was lower than that in the control group, suggesting that hypoxia diminished the hepatic differentiation ability of iHepSCs (Supplemental Figure 1A).

Compared with prolonged hypoxia exposure mentioned above, the method of short-term hypoxia preconditioning was reported to improve chondrogenic potential of MSCs [31, 32]. To acquire optimal hepatic induction conditions, iHepSCs were then hypoxia-preconditioned, which meant that iHepSCs were firstly cultured under hypoxia for 24 h, and then transferred to normal atmosphere for hepatic induction. Cells in the control group were expanded and hepatic-differentiated in normal conditions. It was found that the expression of hepatocyte-related genes TAT, Ttr, Hnf4a, Hnf1a, Alb, and Afp in the hypoxia preconditioning group was remarkably higher than those in the control group (Supplemental Figure 1B). Besides, ELISA showed that Alb in secretome of hypoxia preconditioning iHepSCs was significantly higher than that in the control group (Figure 4(e)). Furthermore, periodic acid-Schiff (PAS) staining indicated significant glycogen storage as mature hepatocytes, and hypoxia preconditioning group showed higher PAS staining density, indicating that more hepatocytes-like cells were yielded (Figure 4(f)). DiI-labeled acetylated low-density lipoprotein (DiI-ac-LDL) and indocyanine green (ICG) uptake revealed that the induced cells gained the key function of hepatocytes, and hypoxia preconditioning iHepSCs showed a higher percentage of LDL^+^ and ICG^+^ cells (Figures 4(g) and 4(h)). iHepSCs that were not induced to hepatic differentiation were all negative for these three indexes (Supplemental Figure 2). These results indicate that hypoxia preconditioning was a better method to induce differentiation of iHepSCs into hepatocytes efficiently.

## 4. Discussion

Hypoxia is known to be involved in regulating multiple biobehaviors of adult stem cells and induced pluripotent stem cells (iPSCs), including stemness preservation, proliferation, differentiation, metabolism, and aging. However, disparate stem cells reside in distinct microenvironments with different oxygen concentrations in vivo, indicating that the regulating roles of hypoxia on stem cells are diverse. 1% O_2_ promotes the proliferation of neural progenitors and enhances their clonal growth [33]. The optimal oxygen concentration for MSC expansion is 5% [7, 32]. The liver receives a dual blood supply and is regarded as an oxygen-sufficient organ. Knowing that the oxygen concentration in the periportal area where liver stem cells (or liver progenitors) reside is as high as 9–11%, we chose 10% O_2_ to culture iHepSCs. Consistent with our hypothesis, physiological hypoxia not only enhanced the stemness of iHepSCs but also promoted their expandable ability. Actually, we tried an extreme hypoxic condition (1% O_2_), but iHepSCs lost the stemness properties and presented diminished expansion (Supplemental Figure 3). These results strongly suggest that 10 O_2_ is a better cultural condition for quick expansion of iHepSCs. Noteworthily, due to the limitation of the hypoxic culture system employed that the medium shift and passage of cells could only be operated outside where is normoxia environment, iHepSCs could not be cultured in hypoxia permanently, so prolonged effects of hypoxia on growth of iHepSCs are unknown and need advanced cultural system with closed operating chamber.

To uncover the mechanism underlying the effect of hypoxia in promoting proliferation of iHepSCs, we focused on cell cycle regulation. Knowing that G1/S transition is one of the most important checkpoints during the cell proliferation process, hypoxia could accelerate G1/S transition, and HIF1a and HIF2a played a key role in this regulation [34, 35], we firstly detected the expression of HIF1a and HIF2a by immunofluorescence staining and Western blot and found that HIF1a was transiently upregulated, and HIF2a showed a constant high expression throughout the hypoxia process. In addition, more hypoxia-cultured iHepSCs were found to be in S phase and fewer ones in G1 phase, suggesting that hypoxia quickened the G1-S entry of iHepSCs. p53-p21 signaling regulates the cell cycle progression in mammalian cells by two downstream effectors (CDK4/6-Cyclin D1 and CDK2-Cyclin E complexes) [36]. Data obtained from the present study showed that p53 and p21 in hypoxia-cultured iHepSCs were inhibited and CDKs were upregulated, which meant that G1/S entry was accelerated via p53-p21 pathway.

Hypoxia plays distinct roles in regulating inducible differentiation of stem cells. MSCs were easier to differentiate into osteoblasts, adipocytes, and chondrocytes under hypoxia [37]. However, other studies found that hypoxia inhibited their chondrogenic differentiation [38]. These inconsistent results may be explained by the following reasons. First, cell populations from different sources and tissues are heterogeneous. Second, the inducing methods are various. Third, the degree of hypoxia is diverse [38]. Our study showed that 10% O_2_ culture facilitated cholangiocytic differentiation in iHepSCs while hepatic differentiation was inhibited that may be because cholangiocytic and hepatic differentiation shared competitive signaling pathways, and hypoxia-cultured iHepSCs shared more gene identities with cholangocytes than with hepatocytes. However, to our surprise, when iHepSCs were preconditioned in hypoxia for 24 h and then transferred to normal conditions for hepatic induction, the efficiency was significantly and remarkably improved. Actually, it has been reported that hypoxia is required for quick expansion of MSCs but not for committed differentiation [38]. It is noteworthy that hypoxic preconditioning enhanced chondrogenic differentiation but decreased osteogenic differentiation in mouse adipose stromal cells (ASCs) [39]. Additionally, rejuvenation of stem cells is essential for constant expansion and maintenance of multidifferentiation potential [40]. After hypoxia preconditioning for 24 h, iHepSCs expressed less p53 and p21, two main senescence-related proteins, which might benefit the hepatic differentiation. However, the exact mechanism needs to be further elucidated.

## 5. Conclusion

Physiological hypoxia (10% O_2_) could enhance the stemness properties and augment the yield of iHepSCs by promoting the proliferation ability via accelerating G1/S transition. Moreover, the hypoxia condition promoted Matrigel-induced cholangiocytic differentiation but inhibited hepatic differentiation of iHepSCs. Interestingly, short-term hypoxia preconditioning endows iHepSCs with enhanced hepatic differentiation potential. These results to some extent reveal the effects and mechanism of hypoxia on biological behaviors of iHepSCs and show promising perspective to explore optimal culture conditions for therapeutic stem cells.

## Supplementary Materials

Supplementary 1 Supplemental Figure 1: effects of hypoxia and hypoxia preconditioning on hepatic differentiation of iHepSCs.

Supplementary 2 Supplemental Figure 2: PAS staining, DiI-ac-LDL uptake, and indocyanine green (ICG) uptake: all negative before induction of iHepSCs to hepatocytes.

Supplementary 3 Supplemental Figure 3: the proliferation ability and stemness properties of iHepSCs under normoxia and extreme hypoxic condition.

Supplementary 4 Supplemental Table 1: the primer list.

Supplementary 5 Supplemental Table 2: primary and secondary antibodies.
