# Supplementary material for: Physiological Hypoxia Enhances Stemness Preservation, Proliferation, and Bidifferentiation of Induced Hepatic Stem Cells
Source: Oxid Med Cell Longev. 2018 Feb 13;2018:7618704. doi: 10.1155/2018/7618704 (PMC5831960; doi:10.1155/2018/7618704)
Supplement: Supplementary 1 — Supplemental Figure 1: effects of hypoxia and hypoxia preconditioning on hepatic differentiation of iHepSCs. [file 7618704.f1.docx]

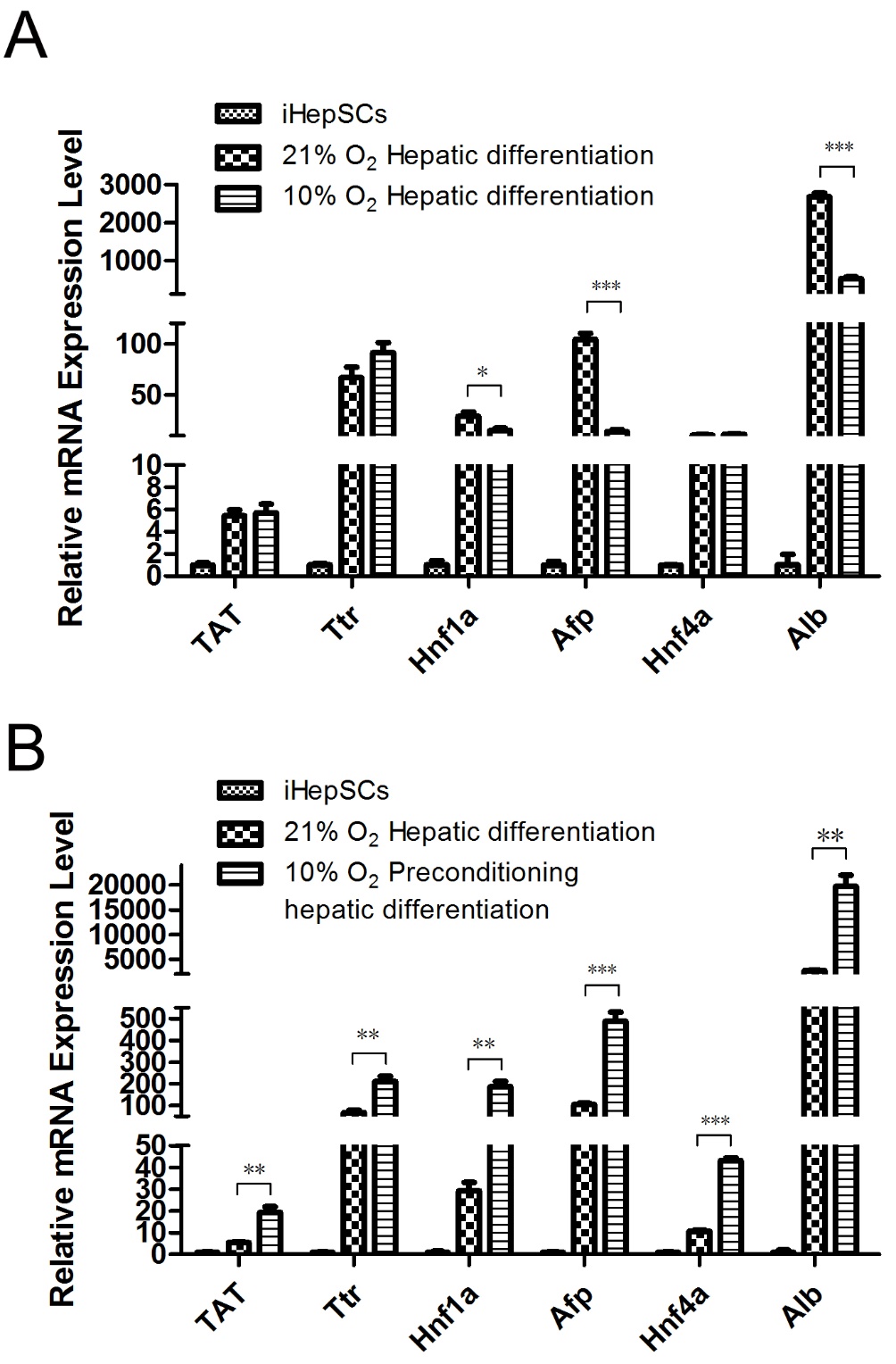


Supplemental Fig. 1. Effects of hypoxia and hypoxia preconditioning on hepatic differentiation of iHepSCs. (A) qRT-PCR: relative expression of TAT, Ttr, Hnf4a, Hnf1a, Alb and Afp in the control and hypoxic groups. (B) qRT-PCR: relative expression of TAT, Ttr, Hnf4a, Hnf1a, Alb and Afp in the control and hypoxia-preconditioning groups. Statistical significance: * p<0.05, ** p<0.01, *** p<0.001.
