# Supplementary material for: Physiological Hypoxia Enhances Stemness Preservation, Proliferation, and Bidifferentiation of Induced Hepatic Stem Cells
Source: Oxid Med Cell Longev. 2018 Feb 13;2018:7618704. doi: 10.1155/2018/7618704 (PMC5831960; doi:10.1155/2018/7618704)
Supplement: Supplementary 2 — Supplemental Figure 2: PAS staining, DiI-ac-LDL uptake, and indocyanine green (ICG) uptake: all negative before induction of iHepSCs to hepatocytes. [file 7618704.f2.docx]

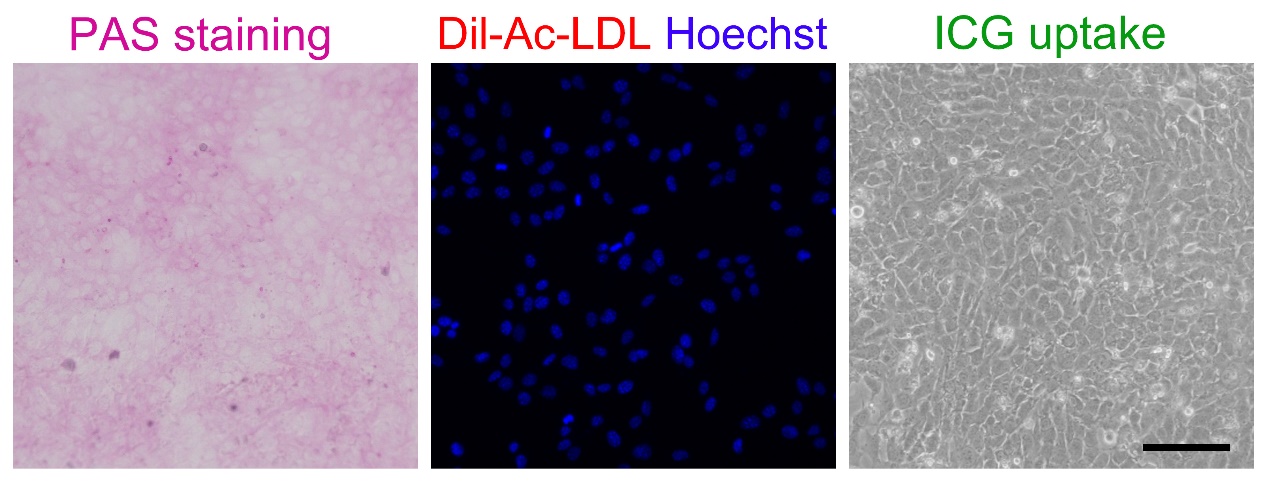


Supplemental Fig. 2. PAS staining, DiI-ac-LDL uptake, and indocyanine green (ICG) uptake: all negative before induction of iHepSCs to hepatocytes. Scale bars=100μm.
