# Supplementary material for: Physiological Hypoxia Enhances Stemness Preservation, Proliferation, and Bidifferentiation of Induced Hepatic Stem Cells
Source: Oxid Med Cell Longev. 2018 Feb 13;2018:7618704. doi: 10.1155/2018/7618704 (PMC5831960; doi:10.1155/2018/7618704)
Supplement: Supplementary 3 — Supplemental Figure 3: the proliferation ability and stemness properties of iHepSCs under normoxia and extreme hypoxic condition. [file 7618704.f3.docx]

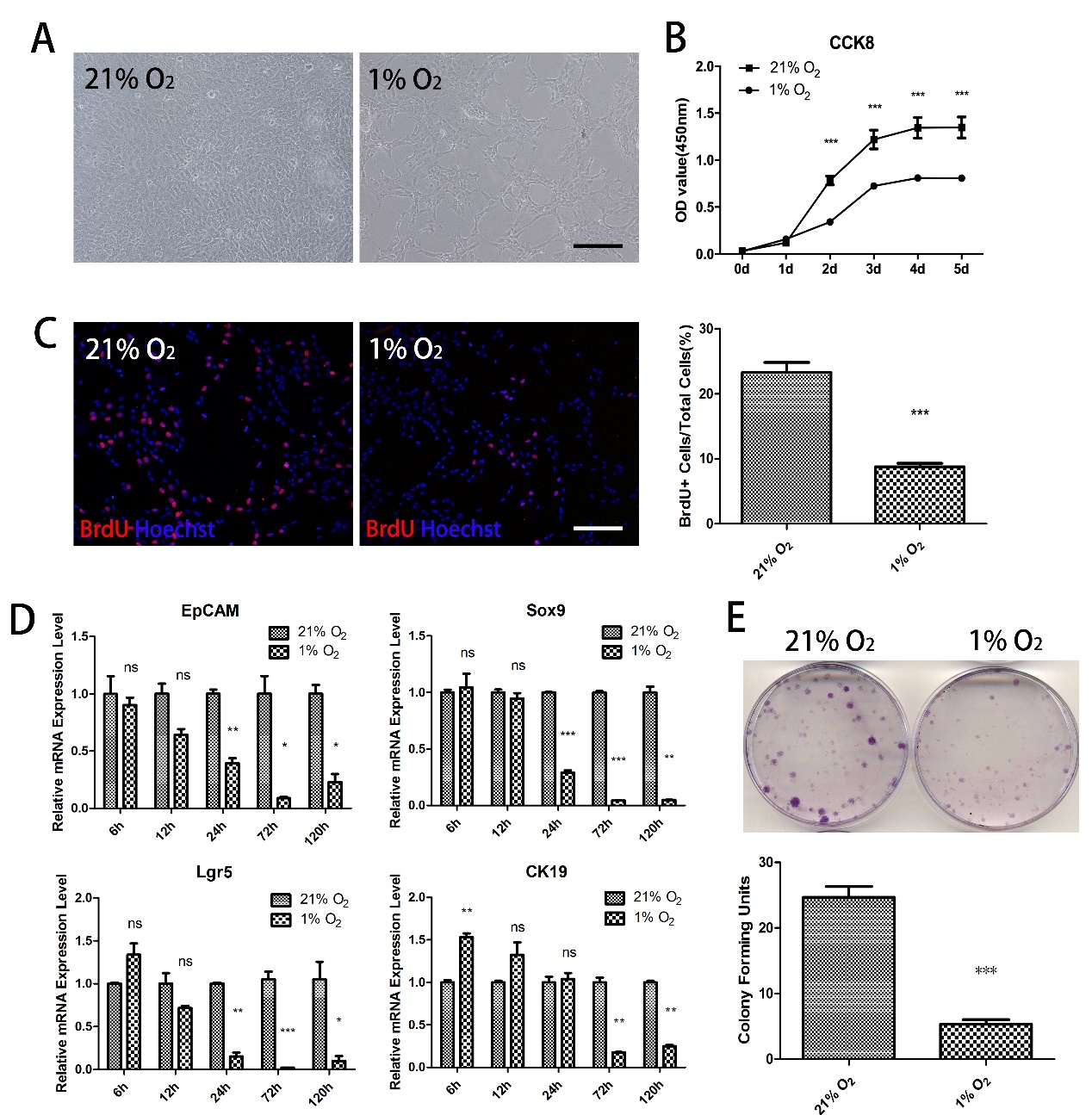


Supplemental Fig. 3. The proliferation ability and stemness properties of iHepSCs under normoxia and extreme hypoxic condition. (A) Morphology of iHepSCs under the bright field in each group. (B) CCK8 assay: proliferation kinetics of iHepSCs cultured under normoxia and that under extreme hypoxia. (C) BrdU incorporation and quantitative analysis: ratio of BrdU-positive cells to total cells in each group. (D) Relative expression of stem cell markers of iHepSCs cultured in normoxia and extreme hypoxia during the time course. (E) Colony forming units and quantitative analysis of each group. Statistical significance: * p<0.05, ** p<0.01, *** p<0.001. Scale bars=100μm.
