# Supplementary material for: Physiological Hypoxia Enhances Stemness Preservation, Proliferation, and Bidifferentiation of Induced Hepatic Stem Cells
Source: Oxid Med Cell Longev. 2018 Feb 13;2018:7618704. doi: 10.1155/2018/7618704 (PMC5831960; doi:10.1155/2018/7618704)
Supplement: Supplementary 4 — Supplemental Table 1: the primer list. [file 7618704.f4.docx]

**Supplemental Table1. The primer list**

| Gene | Forward  (5’→3’) | Reverse  (5’→3’) | Tm  (°C) | Length  (bp) |
| --- | --- | --- | --- | --- |
| TAT | GCTTCACCAGGTTAATGAGAAGCT | TCCGCAATTAACCGCTCTGT | 60 | 223 |
| Abcg2 | TAAATGGAGCACCTCAACCTG | GGATAAACTGAGTTCCGACCT | 57 | 228 |
| Alb | AAAGCATGGGCAGTAGCTCG | AGTCGCCTGGTTTTCACACA | 60 | 177 |
| CK19 | CCCTCCCGAGATTACAACCAC | CCTCCACGCTCAGACGCAAG | 57 | 175 |
| Hnf1a | CCACAGAGCTTGACTAGTGGG | TAGAAACCATGGCTCCGCTG | 60 | 164 |
| GAPDH | GGTTGTCTCCTGCGACTTCA | TCTTGCTCAGTGTCCTTGCT | 58 | 211 |
| Ggt1 | ACCATCTACAACAGCACCAC | GCTCATAACCACGGATTTCACC | 56 | 154 |
| Gja1 | GCGGATCTCCAAAATATGCTT | CCCCATTCGATTTTGCTCT | 56 | 185 |
| Hnf4a | TAGGCAATGACTACATCGTCCC | CGGTCGTTGATGTAATCCTCC | 58 | 244 |
| Afp | AGTTTCCAGAACCTGCCGAG | ACCTTGTCGTACTGAGCAGC | 60 | 145 |
| Ttr | GCCTCGCTGGACTGGTATTT | GGTCTTCCCAGAGGCAAAGG | 60 | 179 |
