# Supplementary material for: Physiological Hypoxia Enhances Stemness Preservation, Proliferation, and Bidifferentiation of Induced Hepatic Stem Cells
Source: Oxid Med Cell Longev. 2018 Feb 13;2018:7618704. doi: 10.1155/2018/7618704 (PMC5831960; doi:10.1155/2018/7618704)
Supplement: Supplementary 5 — Supplemental Table 2: primary and secondary antibodies. [file 7618704.f5.docx]

**Supplemental Table2. Primary and secondary antibodies**

**Primary antibodies**

| Antibody | Catalog | Source | Dilution | Company |
| --- | --- | --- | --- | --- |
| HIF1a | NB100-479 | Rabbit polyclonal | 1:2000 | Novus Biologicals |
| HIF2a | NB100-122 | Rabbit polyclonal | 1:1000 | Novus Biologicals |
| BrdU | Ab6326 | Rat monoclonal | 1:200 | Abcam |
| EpCAM | ab32392 | Rabbit polyclonal | 1:200 | Abcam |
| EpCAM | ab212580 | Mouse monoclonal | 1:200 | Abcam |
| Lgr5 | ab75732 | Rabbit polyclonal | 1:200 | Abcam |
| Sox9 | AB5535 | Rabbit polyclonal | 1:500 | Millipore |
| GAPDH | 5174S | Rabbit polyclonal | 1:3000 | CST |
| P21 | Ab7960 | Rabbit polyclonal | 1:1000 | Abcam |
| P53 | Ab26 | Mouse monoclonal | 1:1000 | Abcam |
| CDK2 | Sc-163 | Rabbit polyclonal | 1:1000 | Santa Cruze |
| CDK4 | Sc-260 | Rabbit polyclonal | 1:1000 | Santa Cruze |
| CDK6 | 14052-1 | Rabbit polyclonal | 1:1000 | Proteintech |
| CyclinD1 | MA1033 | Mouse monoclonal | 1:1000 | Boster |
| CyclinE | BA0774 | Rabbit polyclonal | 1:1000 | Boster |
| Krt19 | A3190 | Rabbit polyclonal | 1:400 | Abbomax |
| Albumin | Ab19194 | Rabbit polyclonal | 1:200 | Abcam |

**Secondary antibodies**

| Antibody | Catalog | Dilution | Company |
| --- | --- | --- | --- |
| Alexa Fluor® 488 Donkey Anti-Rabbit IgG | A21206 | 1:500 | Molecular  Probes |
| Alexa Fluor® 555 Goat Anti-Rat IgG | A21434 | 1:500 | Molecular  Probes |
| Alexa Fluor® 555 Goat Anti-Mouse IgG | A32727 | 1:500 | Molecular  Probes |
| Alexa Fluor® 568 Donkey Anti-Rabbit IgG | A10042 | 1:500 | Molecular  Probes |
| HRP-Goat anti-Mouse IgG | 115-005-003 | 1:4000 | Jackson |
| HRP-Goat anti-Rabbit IgG | 111-035-144 | 1:4000 | Jackson |
